# Explaining withdrawal’s persistence: correlates of withdrawal use in Albania, Armenia, Jordan, and Turkey observed in a cross-sectional study

**DOI:** 10.12688/gatesopenres.13295.1

**Published:** 2021-06-09

**Authors:** Timothee Fruhauf, Ghada Al-Attar, Amy O. Tsui

**Affiliations:** 1 Gynecology and Obstetrics, Johns Hopkins University School of Medicine, Baltimore, Maryland, 21287, USA; 2 Public Health and Community Medicine, Assiut University, Assiut, 71515, Egypt; 3 Population and Reproductive Health, Johns Hopkins Bloomberg School of Public Health, Baltimore, Maryland, 21205, USA

**Keywords:** Withdrawal, Family Planning, Contraception, Albania, Armenia, Jordan, Turkey

## Abstract

**Background: **Withdrawal dominates the contraceptive method mix in a geographical cluster of countries in South-Eastern Europe and Western Asia that have, in part, reached low fertility. This study examines the socio-demographic determinants associated with withdrawal use in Armenia, Albania, Jordan and Turkey that could explain withdrawal’s persistence and inform contraceptive programs in these unique settings.

**Methods: **Cross-sectional data on 31,569 married women 15 to 49 years were drawn from the Demographic and Health Surveys in Albania (2017-2018), Armenia (2015-2016), Jordan (2017-2018), and Turkey (2013). For each country, multinomial regression models estimating withdrawal use among all women and logistic regression models estimating withdrawal use among contraceptive users were used to evaluate the association with age, marital duration, parity, education, residence, and household wealth.

**Results: **The socio-demographic determinants associated with withdrawal use varied by country among all women and among all contraceptive users. While these associations were not all significant for all four countries general trends included that women were more likely to use withdrawal than not use contraception, but less likely to use withdrawal than other methods with increasing parity, higher education, and greater household wealth. Measures of association are reported by country for each correlate.

**Conclusions: **Despite the similar contraceptive mix in these four countries, no single set of factors was found to explain withdrawal’s persistence. Withdrawal’s prevalence in this geographical cluster may instead result from different balances of intertwined circumstances that include couples’ fertility decisions, access to modern contraception and availability of abortion services.

## Introduction

Withdrawal or coitus interruptus is one of the most widely used male contraceptive methods in the world ^1– 3^. Withdrawal is often mentioned for its role in facilitating fertility declines in North America and Northern Europe prior to the introduction of modern contraceptives ^1, 4, 5^. However, it remains an important contributor to the composition or mix of contraceptive methods used in some areas that have reached low fertility ^1, 3, 6^.

Considered a traditional method, withdrawal is frequently grouped with less-effective folkloric methods and viewed as a transitional method or as an alternative of last resort when the only other option is to not use contraception ^2, 6^. Discussions of this method often adopt a problem-oriented approach and assume withdrawal’s low effectiveness translates to low desirability among users ^5, 7^. Yet effectiveness studies stem mostly from North America and may not be representative of societies where withdrawal is extensively and consistently practiced ^8^. Furthermore, the persistence of withdrawal in many societies despite growing access to modern methods points to a more nuanced narrative.

**Figure 1.**
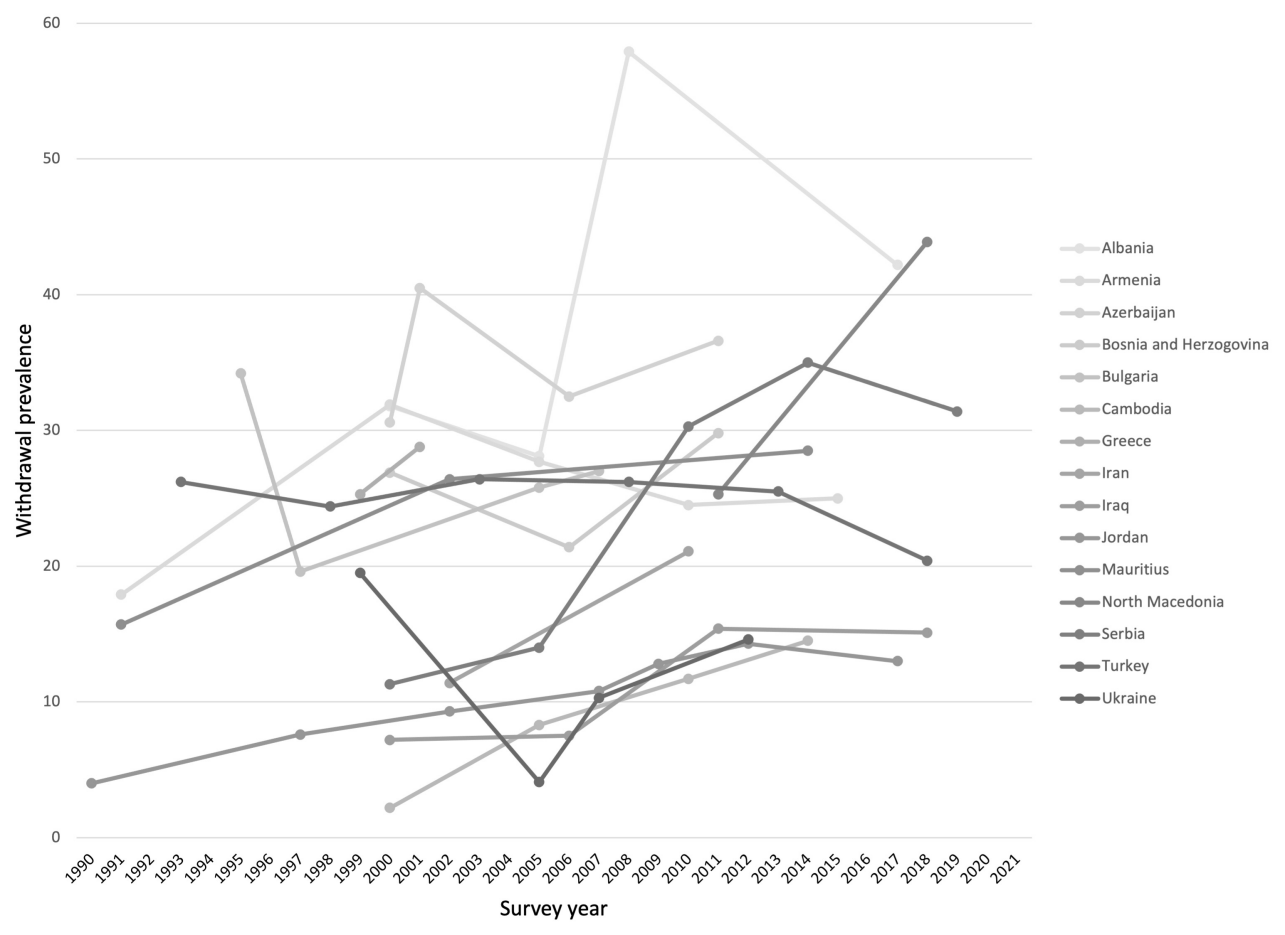
Prevalence of withdrawal use between 1990 and 2021 in 15 countries with the highest prevalence in 2021, as reported by currently married women 15 to 49 years.

A cluster of countries in South-Eastern Europe and Western Asia consistently reports high withdrawal prevalence up to 43.9% among married women of childbearing age in North Macedonia when considering the last ten years of public data ( Table 1) ^3^. The persistence of withdrawal use over time is particularly notable as evidenced by Figure 1 which depicts the prevalence of withdrawal use between 1990 and 2021 in the 15 countries with the highest prevalence in 2021. Studies focusing on factors to explain this exceptional birth control pattern point to women’s and men’s contraceptive competence, previous experience and evaluation of other methods as key drivers of withdrawal use. Fear of side effects and adverse health consequences from modern contraceptives, as well as preference for a natural, convenient, and free method are often cited ^4, 9– 13^. Other researchers highlight cultural norms including interpretation of withdrawal as a mark of discipline and self-control in certain communities and the belief that withdrawal is as effective as modern methods ^11, 14, 15^. There is little known about the factors that could explain the persistence of withdrawal use in this region.

**Table 1.** Most recent contraceptive and withdrawal prevalence and year of most recent available data for countries in the geographic withdrawal cluster of Southern Europe, Eastern Europe, and Western Asia.

Country	Contraceptive prevalence	Withdrawal prevalence	Withdrawal Proportion of Contraceptive Prevalence	Most recent data (year)
*Southern Europe*				
**Albania**	**46.0**	**42.2**	**91.7**	**2017-18**
Andorra	NA	NA	NA	NA
Bosnia and Herzegovina	45.8	29.8	65.1	2011-12
Croatia	58.0	47.6	82.1	1970
Gibraltar	NA	NA	NA	NA
Greece	76.2	28.8	37.8	2001
Holy See	NA	NA	NA	NA
Italy	62.7	18.2	29.0	1995-96
Malta	85.8	40.6	47.3	1993
Montenegro	20.7	5.7	27.5	2018
North Macedonia	59.9	43.9	73.3	2018-19
Portugal	86.8	3.5	4.0	2006
San Marino	NA	NA	NA	NA
Serbia	62.3	31.4	50.4	2019
Slovenia	78.9	8.0	10.1	1994
Spain	62.1	0.9	1.4	2018
*Eastern Europe*				
Belarus	71.2	6.1	8.6	2017
Bulgaria	69.2	27.0	39.0	2007
Czechia	72.0	7.3	10.1	1997
Hungary	61.6	4.8	7.8	2008-09
Poland	62.3	9.0	14.4	2014
Republic of Moldova	56.0	10.3	18.4	2020
Romania	69.8	6.1	8.7	2005
Russian Federation	68.0	12.0	17.6	2011
Slovakia	NA	NA	NA	1997
Ukraine	65.4	14.6	22.3	2012
*Western Asia*				
**Armenia**	**57.1**	**25.0**	**43.8**	**2015-16**
Azerbaijan	54.9	36.6	66.7	2011
Bahrain	61.8	26.3	42.6	1995
Cyprus	NA	NA	NA	NA
Georgia	40.6	3.2	7.9	2018
Iraq	52.8	15.1	28.6	2018
Israel	68.0	11.0	16.2	1987-88
**Jordan**	**51.8**	**13.0**	**25.1**	**2017**
Kuwait	52.0	5.7	11.0	1999
Lebanon	54.5	5.1	9.4	2009
Oman	29.7	8.7	29.3	2014
Qatar	37.5	1.2	3.2	2012
Saudi Arabia	24.6	0.9	3.7	2016
State of Palestine	57.3	9.8	17.1	2019-20
Syrian Arab Republic	58.3	1.7	2.9	2006
**Turkey**	**69.8**	**20.4**	**29.2**	2018 *
United Arab Emirates	27.5	1.4	5.1	1995
Yemen	33.5	2.6	7.8	2013

*2018 Turkey data are not in the public domain. Most recent DHS data are from 2013. The four countries included in this study are bolded. Abbreviations: DHS: Demographic and Health Surveys, NA: not available Source: United Nations, Department of Economic and Social Affairs, Population Division (2021). World Contraceptive Use 2021 (POP/DB/CP/Rev2021).

The literature often depicts withdrawal as a homogeneous phenomenon whereby cultural factors are the primary mediators of the practice. The geographical clustering of these countries in South-Eastern Europe and Western Asia undoubtedly supports the impact of common cultural factors. A few studies exploring the effect of socio-demographic variables on withdrawal use provide conflicting results and point away from a single narrative for all countries. For example, while one study in Iran found that younger, well educated women in urban areas were more likely to use withdrawal, another study also in Iran showed that wealthier, more educated, older women with lower parity preferred withdrawal ^14, 16, 17^. In Turkey, withdrawal use was highest among poorer, less educated, older women with lower parity in rural areas ^17^. To date, this mixed evidence has not been reconciled and its relevance to countries with the highest and most persistent withdrawal use in the South-Eastern Europe and Western Asia belt is unknown.

In addition, while withdrawal’s prevalence in this cluster has undoubtedly allowed many women to achieve their fertility intentions, there remains an unmet need for modern contraception in these countries. Given their unique contraceptive mix at present, that need may only be bridged through unique interventions tailored to these profiles. The aim of this study is to identify the socio-demographic determinants of withdrawal use compared to those not using contraception and those using other methods in four countries of the South-Eastern Europe and Western Asia belt. This information would not only provide additional explanations to the persistence of withdrawal use, but also expand the fulfillment of women’s reproductive goals in this setting.

## Methods

### Sample

**Figure 2.**
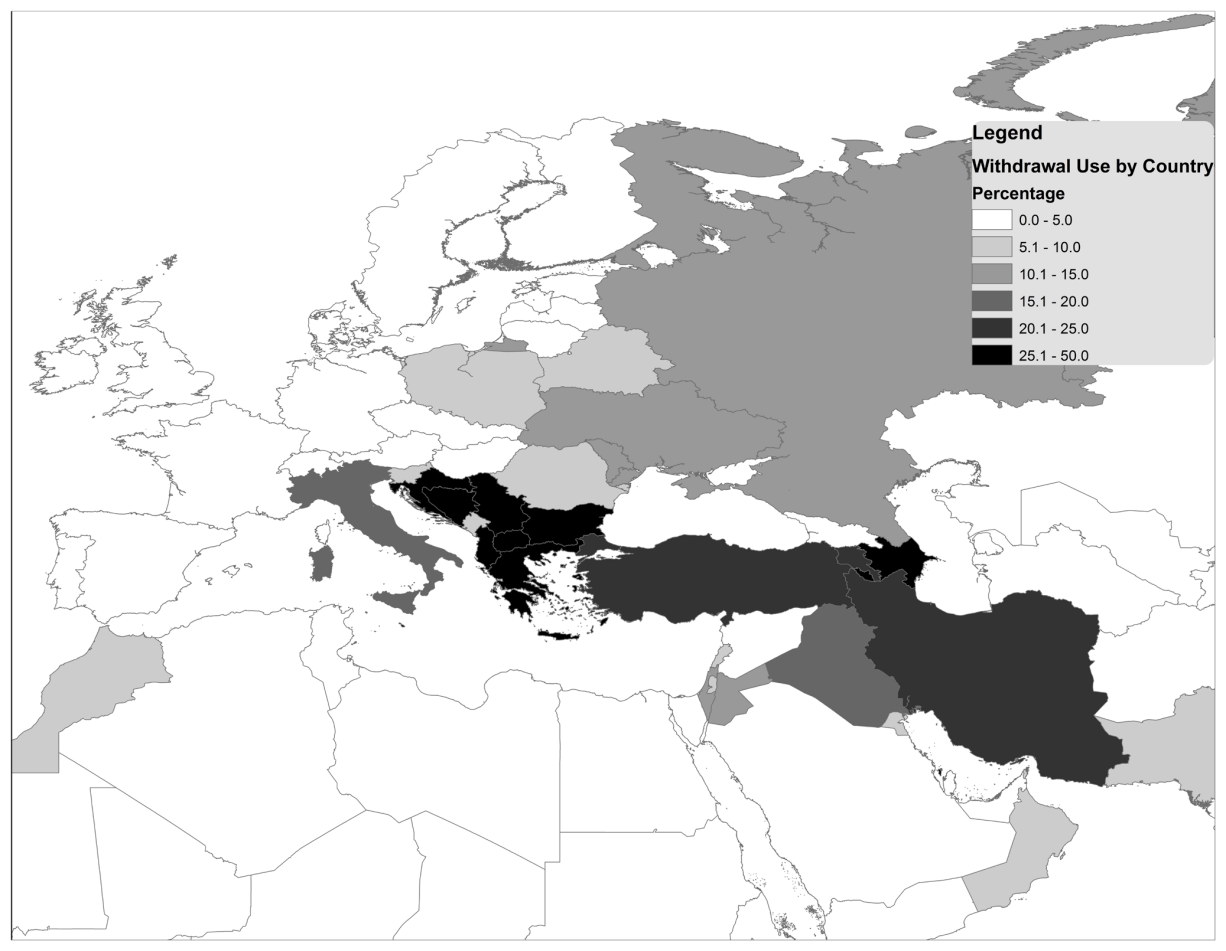
Most recent withdrawal prevalence in South-Eastern Europe and Western Asia.

**Figure 3.**
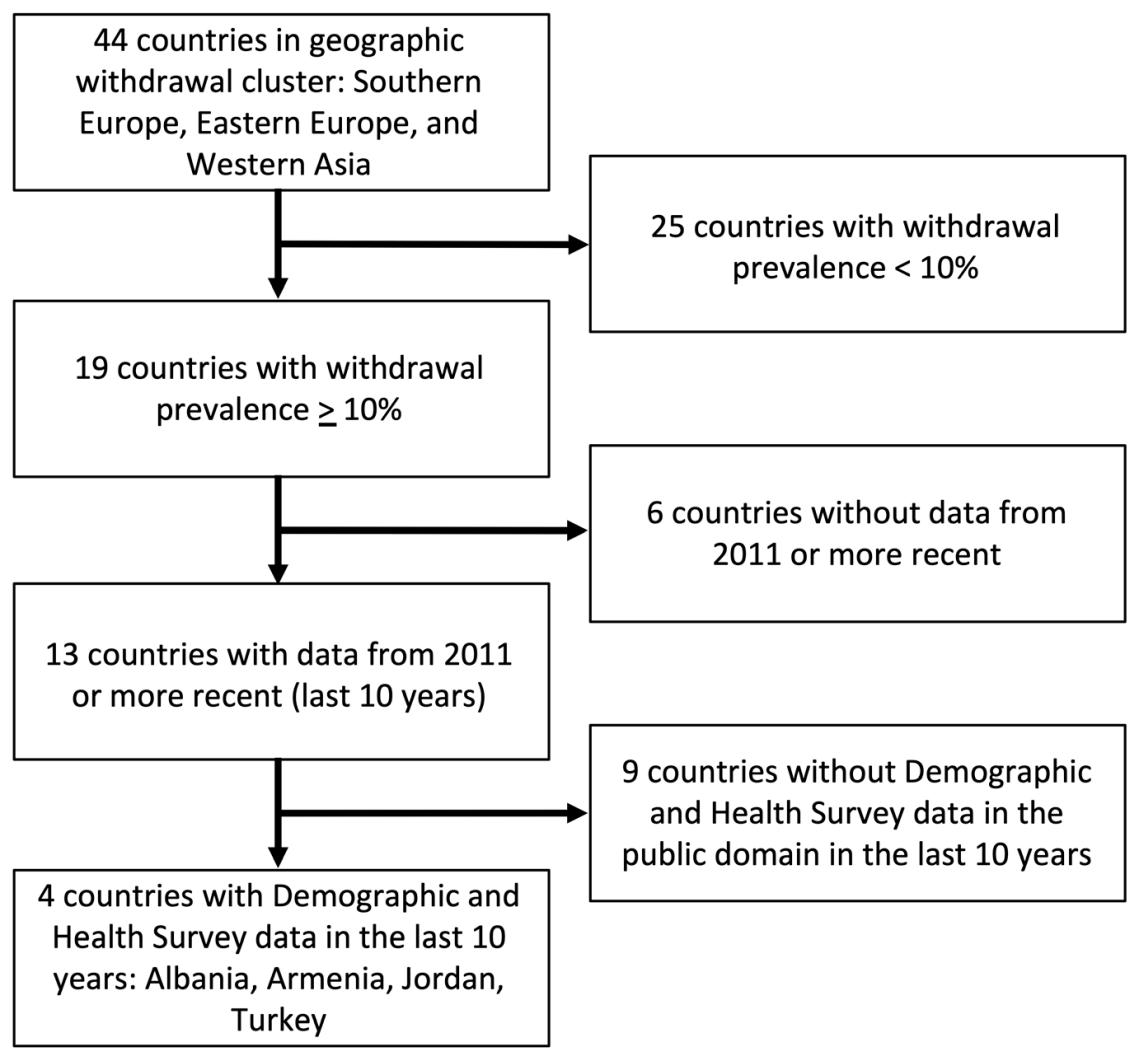
Selection of four study countries.

Withdrawal use among childbearing aged women in union, as reported by the United Nations World Contraceptive Use 2021, was noted to be geographically clustered in three regions defined by the United Nations Statistics Division and made up of 44 countries: Southern Europe, Eastern Europe, and Western Asia ( Figure 2) ^3^. In these regions, withdrawal prevalence ranges from 0.9% (Saudi Arabia and Spain) to 43.9% (Serbia), when considering data less than 10 years old, and represents between 1.4% (Spain) and 91.7% (Albania) of the contraceptive method mix in these countries ( Table 1). All countries in the geographic cluster with a withdrawal prevalence ≥ 10% and with publicly accessible Demographic and Health Survey (DHS) data less than 10 years old were included in this analysis: Albania 2017–18 ^18^, Armenia 2015–16 ^19^, Jordan 2017–18 ^20^, and Turkey 2013 ^21^. Figure 3 outlines the selection process for the countries in this analysis. The DHS surveys are nationally representative household surveys with a cross sectional design and multiple questionnaires. This analysis utilizes data collected through the Woman’s Questionnaire. For each country, women ages 15 to 49 who were married or in union were the units of analysis. Survey data were weighted according to their complex sampling designs for the analysis.

### Variables

The outcome of interest was current contraceptive method use with a focus on withdrawal use. Although the focus is on a male-practiced method, this study relied on female respondents’ report of their partners’ behavior and characteristics which could differ from males’ reports. However, male respondents were not included in the surveys of all countries of interest and were not queried on contraceptive use using the same survey questions as used for women. The first analysis focused on withdrawal use among all married women and the outcome was categorized as no contraceptive method use, withdrawal use, or use of any other contraceptive method. The second analysis, restricted to contraceptive users, categorized the outcome as withdrawal use or use of another method. Demographic and socioeconomic covariates typically associated with family planning use were included: the continuous variables were ages of the respondent and her partner, number of living children, and marital duration, the categorical variables were highest education levels of the respondent and her partner (categorized as primary or no education, secondary education, or higher), urban or rural residence, and household wealth quintile. Household wealth is an asset-based measure which is scored following a factor analysis and divided into five groups that represent the poorest to the richest quintiles.

### Statistical analyses

Multinomial logistic regression models were used to estimate the adjusted relative risk ratios (RRR) of withdrawal use and use of other methods compared to no contraceptive use. Logistic regression models were used to estimate the adjusted odds ratio (aOR) of withdrawal use compared to use of other methods. Models were adjusted for covariates described above. Statistical significance for alpha of 0.05 and 0.01 are reported. Analyses were conducted in Stata 15 (College Station, TX: StataCorp LP).

## Results

Withdrawal prevalence ranged between 13.0% in Jordan and 42.2% in Albania among the four study countries ( Table 2). Withdrawal was the most commonly used method in all countries except Jordan and represented between 25.2% (Jordan) and 91.8% (Albania) of the national method mix. Other male methods were not as prevalent in all four countries though male condoms were important in Armenia (14.7%) and Turkey (15.8%). No use of male sterilization was reported by the female respondents in any of the study countries.

**Table 2.** Current contraceptive use by currently married women age 15 to 49 years in 4 study countries.

	Albania	Armenia	Jordan	Turkey
DHS year	2017–2018	2015–2016	2017–2018	2013
Number of women	7403	3895	13616	6655
*Contraceptive prevalence (%)*				
Any method	46.0	57.1	51.8	73.5
Any female method	2.4	17.5	33.7	32.0
Any male method	43.7	39.7	18.1	41.4
Withdrawal	42.2	25.0	13.0	25.5
Condom	1.4	14.7	5.1	15.8
Male sterilization	0.0	0.0	0.0	0.0
Number of users	3406	2226	7057	4888
*Contraceptive method mix (%)*				
Any female method	5.1	30.6	65.0	43.6
Withdrawal	91.8	43.7	25.2	34.8
Other male methods	3.1	25.7	9.9	21.6

The characteristics of the 31,569 married women of reproductive age included in the study are disaggregated by contraceptive use and covariates of interest in Table 3. Withdrawal use was lowest among younger women and their partners in all countries and increased with age in Albania and Armenia. In Armenia and Jordan, and largely in Turkey, withdrawal use increased with the number of children, but the pattern was curvilinear in Albania with highest use at 1-2 children. Except in Turkey, withdrawal use was lowest when marital duration was under 10 years. In all countries except Albania, withdrawal use was more prevalent among rural women. Withdrawal use increased with higher levels of education of women and their partners in Albania and Jordan, while it decreased in Armenia and Turkey. Withdrawal use rose with increasing wealth in Albania, but decreased in Armenia. The relationship pattern was moderately curvilinear in Jordan and Turkey with more use reported by women in the middle household wealth quintiles. The associations between these factors and withdrawal use are further explored in the regression models detailed below.

**Table 3.** Sociodemographic characteristics of currently married women age 15 to 49 years by contraceptive method used in 4 study countries.

	Albania					Armenia					Jordan					Turkey				
	Mean (Standard Error) or Percentage					Mean (Standard Error) or Percentage					Mean (Standard Error) or Percentage					Mean (Standard Error) or Percentage				
	withdrawal N=3126	other methods N=280	non-use N=3997	total	N	withdrawal N=973	other methods N=1253	non-use N=1669	total	N	withdrawal N=1776	other methods N=5281	non-use N=6559	total	N	withdrawal N=1700	other methods N=3189	non-use N=1767	total	N
*Age of the respondent* *(years)*	36.1 (0.2)	34.5 (0.6)	30.3 (0.2)	36.4 (0.1)	7403	35.4 (0.3)	34.1 (0.2)	29.7 (0.2)	34.7 (0.2)	3895	34.9 (0.3)	35.9 (0.2)	34.2 (0.2)	34.7 (0.1)	13616	34.4 (0.3)	35.2 (0.2)	27.0 (0.2)	34.7 (0.1)	6655
<25	34.8	3.3	61.9	10.0	739	17.3	24.4	58.3	10.2	398	10.6	21.2	68.2	13.3	1813	25.8	28.5	45.7	11.0	730
25–34	40.2	4.1	55.7	31.2	2311	24.1	36.8	39.1	40.7	1586	14.2	37.5	48.4	35.9	4892	26.7	48.9	24.4	38.5	2560
35–44	47.3	4.6	48.1	36.5	2704	28.5	35.2	36.3	34.6	1348	13.4	47.8	38.8	34.9	4748	25.2	56.9	17.9	37.2	2478
45+	40.1	2.1	57.8	22.3	1649	24.3	17.4	58.3	14.4	562	11.9	36.7	51.4	15.9	2164	23.0	36.0	41.0	13.3	888
*Age of the partner* * (years)*	42.4 (0.3)	41.7 (0.6)	41.8 (0.2)	42.1 (0.2)	7403	40.2 (0.3)	38.7 (0.2)	39.3 (0.3)	39.3 (0.2)	3894	40.2 (0.4)	41.6 (0.2)	39.7 (0.2)	40.5 (0.1)	13616	38.7 (0.3)	39.5 (0.2)	38.4 (0.3)	39.0 (0.2)	6651
<25	38.8	3.8	57.4	2.0	145	14.6	18.6	66.8	2.7	107	7.5	15.8	76.7	3.3	445	20.3	19.5	60.2	2.6	170
25–34	38.9	2.9	58.2	21.3	1575	20.3	33.5	46.2	31.2	1215	13.2	31.0	55.8	26.9	3657	27.7	41.6	30.7	31.5	2093
35–44	43.3	4.7	52.0	33.4	2473	27.7	38.6	33.7	34.6	1346	14.4	43.1	42.5	34.8	4732	24.3	56.8	18.8	38.2	2544
45+	43.2	3.5	53.3	43.4	3210	27.6	25.0	47.4	31.5	1226	12.1	42.6	45.3	35.1	4782	25.3	45.4	29.3	27.7	1845
*Marital duration (years)*	15.3 (0.2)	14.1 (0.6)	14.9 (0.2)	15.0 (0.1)	7403	15.2 (0.3)	13.0 (0.2)	13.9 (0.3)	13.6 (0.2)	3895	13.5 (0.3)	15.0 (0.2)	12.0 (0.2)	13.2 (0.1)	13616	13.8 (0.2)	14.7 (0.2)	13.5 (0.3)	13.9 (0.1)	6655
0–9	39.3	3.5	57.2	32.2	2387	18.7	31.0	50.3	38.1	1485	12.2	27.9	59.9	40.3	5485	25.6	39.6	34.9	36.4	2424
10–19	45.5	4.9	49.6	32.8	2430	28.0	40.7	31.3	32.7	1274	14.2	47.5	38.3	32.6	4437	25.3	57.0	17.7	35.1	2333
20+	41.9	3.0	55.1	34.9	2585	29.7	24.2	46.2	29.2	1136	12.9	44.4	42.7	27.1	3694	25.8	47.4	26.8	28.5	1898
*Number of living* * children*	1.9 (0.0)	2.0 (0.1)	1.1 (0.0)	2.0 (0.0)	7403	2.3 (0.0)	2.1 (0.0)	0.9 (0.0)	2.0 (0.0)	3895	3.5 (0.1)	3.8 (0.0)	2.3 (0.0)	3.1 (0.0)	13616	2.3 (0.0)	2.5 (0.0)	0.8 (0.0)	2.2 (0.2)	6655
0	24.0	2.9	73.1	8.9	659	1.6	0.8	97.7	6.7	261	0.4	0.5	99.1	11.0	1496	10.4	15.7	73.9	9.7	645
1–2	46.1	2.9	51.0	64.5	4773	24.1	36.1	39.7	69.0	2690	13.6	29.6	56.8	29.3	3986	28.8	48.1	23.1	57.1	3801
3+	39.1	6.1	54.9	26.6	1971	33.8	29.6	36.5	24.2	945	15.1	50.3	34.6	59.7	8134	24.3	57.0	18.7	33.2	2209
*Highest education level, * *Respondent*					7403					3895					13616					6655
No or primary education	40.0	3.5	56.6	49.0	3626	36.3	22.9	40.8	5.4	209	9.7	30.2	60.2	8.9	1212	27.3	45.8	27.0	56.7	3771
Secondary education	43.2	3.2	53.6	30.2	2235	31.0	27.1	41.9	42.9	1669	13.6	40.6	45.8	54.7	7454	25.6	48.8	25.7	31.5	2097
Higher	46.1	5.3	48.6	20.8	1541	18.8	37.3	43.9	51.8	2017	13.0	38.1	48.8	36.4	4950	17.2	55.9	26.8	11.8	787
*Highest education level,* * Partner*					7401					3890					13611					6643
No or primary education	38.6	3.8	57.6	45.8	3390	32.2	22.7	45.1	9.5	371	11.9	32.0	56.1	11.6	1579	26.6	44.5	28.9	40.8	2713
Secondary education	43.9	3.3	52.8	40.1	2964	28.8	28.7	42.5	48.5	1885	13.6	40.0	46.4	57.1	7770	28.1	47.7	24.2	42.5	2822
Higher	49.3	4.9	45.8	14.2	1047	19.0	38.3	42.7	42.0	1634	12.5	39.1	48.4	31.3	4263	16.3	57.0	26.7	16.7	1108
*Residence*					7403					3895					13616					6655
Rural	41.9	3.7	54.4	43.0	3180	32.5	25.6	41.9	43.0	2221	16.7	36.4	46.9	89.7	12214	28.2	40.3	31.5	19.7	1314
Urban	42.5	3.8	53.7	57.0	4223	19.3	37.2	43.5	57.0	1674	12.6	39.1	48.3	10.3	1402	24.9	49.8	25.3	80.3	5341
*Household wealth* *quintile*					7403					3895					13616					6655
Poorest	38.1	3.7	58.2	20.4	1513	34.7	24.0	41.4	17.8	695	12.3	35.4	52.3	19.8	2698	25.8	38.3	35.9	15.6	1038
Poorer	41.6	3.7	54.7	20.9	1550	29.6	27.2	43.2	21.4	834	13.6	37.9	48.5	21.1	2868	28.1	42.5	29.5	19.5	1299
Middle	40.2	3.0	56.8	19.3	1425	26.6	27.8	45.6	18.5	721	15.4	39.7	45.0	20.9	2848	28.3	47.5	24.2	20.5	1366
Richer	40.5	3.7	55.8	19.8	1468	19.6	35.6	44.8	20.3	790	14.4	39.9	45.7	20.8	2835	24.9	52.4	22.7	21.5	1433
Richest	51.1	4.7	44.2	19.5	1446	16.2	44.2	39.6	22.0	855	8.8	41.3	49.9	17.4	2367	21.3	55.2	23.5	22.8	1519

Table 4 examines the association between the sociodemographic correlates and women’s reported use of withdrawal and other methods versus not using contraception. Statistically significant findings are discussed here. Two letter country codes are used to reference specific measures of associations: AL for Albania, AR for Armenia, JO for Jordan, TR for Turkey. In all countries, women were significantly more likely to use withdrawal with increasing number of living children (AL RRR 1.16, AR RRR 2.02, JO RRR 1.62, TR RRR 1.53) than not use contraception. In Armenia, the relative risk for women to use withdrawal was lower with increasing age (RRR 0.96), but greater with increasing marital duration (RRR 1.04). In Jordan, the relative risk for withdrawal use was lower with each rising year of partner age (RRR 0.98). With regards to education, women with higher levels of education had a higher risk ratio to use withdrawal in Jordan (RRRs 2.18, 2.46) and women whose partners had a higher level of education had a higher relative risk for using withdrawal in Albania (RRR 1.43) than for not using contraception. However, in Turkey, women whose partners had a secondary level of education had higher relative risk ratios for using withdrawal (RRR 1.31) than no method. In Albania, Jordan, and Turkey, women from wealthier households compared to poorest were more likely to use withdrawal (AL RRR 1.77, JO RRRs 1.59 and 1.47, TR RRRs 1.66, 2.29, 2.40, 2.54) than not use contraception.

**Table 4.** Relative risk ratios from multinomial logistic regression of withdrawal use and any other method use compared to non-use on selected covariates of married women age 15 to 49 years for 4 study countries.

	Albania	Armenia	Jordan	Turkey
Covariates (Ref = non-use)	RRR	RRR	RRR	RRR
**Use of withdrawal**				
Age of the respondent	1.003	0.957 **	0.994	0.989
Age of the partner	0.999	0.982	0.981 **	0.997
Marital duration	0.998	1.038 **	0.979 *	0.992
Number of living children	1.156 ***	2.018 ***	1.615 ***	1.532 ***
Highest education level, Respondent (ref = no or primary education)				
Secondary education	1.029	0.977	2.182 ***	1.040
Higher	1.061	0.778	2.464 ***	0.882
Highest education level, Partner (ref = no or primary education)				
Secondary education	1.169 *	1.291	1.129	1.310 ***
Higher	1.428 **	1.192	1.162	0.733 **
Residence (ref = rural)	0.822	0.819	0.848	0.834 *
Household wealth quintile (ref = poorest)				
Poorer	1.203 *	0.934	1.240 *	1.663 ***
Middle	1.155	0.987	1.586 ***	2.285 ***
Richer	1.170	0.901	1.469 ***	2.399 ***
Richest	1.766 ***	0.831	0.902	2.536 ***
**Use of any other method**				
Age of the respondent	0.972	0.960 **	0.970 ***	0.980 *
Age of the partner	1.019	0.995	0.990	0.992
Marital duration	0.963 *	0.990	1.000	1.007
Number of living children	1.893 ***	2.201 ***	1.711 ***	1.818 ***
Highest education level, Respondent (ref = no or primary education)				
Secondary education	1.258	1.012	2.142 ***	1.246 **
Higher	2.213 ***	1.198	2.559 ***	1.428 **
Highest education level, Partner (ref = no or primary education)				
Secondary education	0.838	1.479 **	1.323 ***	1.325 ***
Higher	1.000	1.820 ***	1.336 **	1.276 *
Residence (ref = rural)	0.869	1.216	1.163	0.997
Household wealth quintile (ref = poorest)				
Poorer	1.176	1.123	1.247 **	1.818 ***
Middle	0.897	1.015	1.458 ***	2.732 ***
Richer	1.131	1.267	1.378 ***	3.337 ***
Richest	1.716	1.638 ***	1.417 ***	3.652 ***
Observations	7,553	3,992	13,730	6,815

*** p<0.01, ** p<0.05, * p<0.1 Abbreviations: RRR: relative risk ratio, ref: reference

The second panel of Table 4 compares the relative risk for using other contraceptive methods to no method use as associated with the same covariates. While relationship patterns with respondent and partner ages are negative, similar to the patterns for using withdrawal, the relationship with number of children for using any other method is stronger in terms of the magnitude of the relative risk ratios (AL RRR 1.89, AR RRR 2.20, JO RRR 1.71, TR RRR 1.82). The relative risk of using contraception other than withdrawal, over no use, is significantly and strongly associated in Jordan and Turkey with female education (JO RRR 2.14 and 2.56, TR RRR 1.25 and 1.43) and partner education (JO RRR 1.32 and 1.34, TR RRR 1.33 and 1.28). Similarly, the relative risk of using another method versus no contraception significantly increases with household wealth in these two countries. The associations are less consistently significant in Albania and Armenia.

Table 5 focuses on correlates among users, comparing women using withdrawal and those using other methods. In Albania and Jordan, the odds of women using withdrawal were significantly more with increasing age (AL aOR 1.05, JO aOR 1.03) compared to using other methods. The same association is noted in Armenia and Turkey but it is not statistically significant. In Albania, Jordan and Turkey, the odds of women using withdrawal were significantly less likely (AL aOR 0.53, JO aOR 0.91, TR aOR 0.80) than other methods with each additional living child. Increasing marital duration was only significantly associated with the odds of withdrawal use in Armenia (aOR 1.05). In Albania and Turkey, women with higher education had lower odds of using withdrawal (AL aOR 0.46, TR aOR 0.58) compared to women with no/primary education. Armenia and Jordan showed the same association although it was not significant. In Turkey, women’s partners with a higher education (aOR 0.57) had significantly lower odds of using withdrawal than other methods. The association was in the same direction in Armenia and Jordan, but not statistically significant. Women living in urban areas in Armenia and Jordan had lower odds (AR aOR 0.69, JO aOR 0.73) of using withdrawal. The same trend without significance was noted in Albania and Turkey. In Armenia and Jordan, women from the richest households (AR aOR 0.49, JO aOR 0.64) and in Turkey, women from the richer households (aOR 0.74) had lower adjusted odds of using withdrawal compared to women from the poorest households.

**Table 5.** Adjusted odds ratios from logistic regression of withdrawal users compared to non-withdrawal users on selected covariates of married women age 15–49 in 4 study countries.

	Albania	Armenia	Jordan	Turkey
Covariates (Ref = use of non withdrawal methods)	aOR	aOR	aOR	aOR
Age of the respondent	1.045 **	1.004	1.028 **	1.009
Age of the partner	0.975	0.981	0.989	1.005
Marital duration	1.038	1.050 **	0.980 *	0.982 *
Number of living children	0.532 ***	1.002	0.907 **	0.802 ***
Highest education level, Respondent (ref = no or primary education)				
Secondary education	0.782	1.025	1.015	0.812 **
Higher	0.456 ***	0.684	0.940	0.577 ***
Highest education level, Partner (ref = no or primary education)				
Secondary education	1.385 *	0.896	0.860	0.987
Higher	1.339	0.674 *	0.869	0.569 ***
Residence (ref = rural)	0.939	0.688 ***	0.725 ***	0.835 *
Household wealth quintile (ref = poorest)				
Poorer	1.009	0.832	0.993	0.927
Middle	1.340	0.966	1.071	0.855
Richer	1.023	0.694 *	1.055	0.735 **
Richest	1.025	0.492 ***	0.640 **	0.716 *
Observations	3,032	2,272	6,959	4,876

*** p<0.01, ** p<0.05, * p<0.1 Abbreviations: aOR: adjusted odds ratio, ref: reference

## Discussion

In the four study countries, the high demand for withdrawal was matched by couples’ desire to limit childbearing indicating their likely intention to curb fertility with this practice, despite its likely lower effectiveness than other methods (data not shown). In fact, these countries reported between 6.9% (Armenia) and 25.8% (Turkey) of births in the past five years to be unintended or mistimed. While withdrawal dominated the method mix in all countries, those that achieved a higher contraceptive prevalence, had a more diverse mix suggesting a role for modern methods to decrease unmet need. Given that trajectory, this study aimed to further understand the persistent reliance on withdrawal and conversely low adoption of modern methods by investigating the correlates of withdrawal use in four geographically clustered countries in comparison to non-users and users of other methods.

General results are discussed first before examining country-level variations. The first regression model compares users of withdrawal and other methods with non-contracepting women. In general, women with more living children, higher level of education among themselves or their partners, and greater wealth were relatively more likely to use withdrawal than not use a method in multiple countries. The relative association of age, marital duration, and urban/rural residence with withdrawal use varied by country when compared to women not using contraception. By including non-users, this analysis also highlights correlates of contraceptive use when the same associations are noted in both groups of users: women were also more likely to use other methods with increasing number of children, education level and wealth than non-users in certain countries. These findings are in accordance with the general literature on determinants of family planning ^22^. The second model is restricted to users; it compares withdrawal users with users of other methods to more precisely focus on the correlates associated with the selection of withdrawal use over other methods. Overall, having fewer living children and lower socioeconomic status in the form of lower education, rural residence, and lower wealth was associated with higher odds of withdrawal use compared to other methods.

In Albania, women with higher education were more likely to use any method other than withdrawal than not use contraception, but less likely to use withdrawal than other methods compared to women with no/primary education. In fact, women’s knowledge of modern methods was the lowest of these four countries (data not shown) and previous studies report widespread erroneous beliefs in withdrawal’s high effectiveness among users and health care providers ^23, 24^. This limited access to information appears to be playing an important role in Albania’s reliance on withdrawal and may be a remnant of restrictions on contraceptive education during pronatalist communist rule that are slowly being reversed ^25^. Other common barriers such as cultural norms, access, and affordability have not been found to play as important roles in withdrawal’s widespread use and persistence, especially in light of Albania’s developed social marketing sector ^25, 26^.

In Armenia, withdrawal use, in part, can be explained by access; women were more likely to use withdrawal than other methods in rural areas and if they were poorer. Access to modern methods remains restricted to urban settings as a result of a Soviet-inherited centralized health system that has known difficulties with the transition ^27, 28^. Furthermore, availability and several economic disruptions have continued to restrict household spending and limited the development of social marketing programs, supporting evidence that withdrawal may be widely used especially by poorer households because it is free ^27^.

In Turkey and Jordan, unequal access to modern methods also appears to impact withdrawal use: more educated and wealthier women are more likely to use withdrawal than not use contraception, and are less likely to use withdrawal than other methods. These findings are consistent with previous studies of withdrawal in Turkey and Jordan ^4, 9, 10, 15, 17, 29^. Importantly, modern methods are more prevalent and method mixes are more diverse in these two countries than in Albania and Armenia. However, the persistent prevalence of withdrawal reflects not only unequal societies, but possibly traditional enclaves where withdrawal is a practice intertwined with women’s empowerment. Of note, in both Turkey and Jordan having a partner with lower education level was associated with increased withdrawal use compared to other methods. This association could signal, in part, that withdrawal may be the decision of the male partner and possibly more widespread in less egalitarian societies ^30^.

These analyses reveal that no single set of factors can explain the pattern of persistent withdrawal use in these four countries. Rather, some associations highlight that the decision to use withdrawal may result from a couple’s individual risk-benefit analysis weighing not using any contraception against using withdrawal against using another method to achieve their fertility intentions. For example, this study, similar to others, finds that women with fewer children were more likely to use withdrawal than women with more children in Albania, Jordan and Turkey ^15, 17^. The decision to use withdrawal may result from variations in couples’ perceived costs of potential contraceptive failure. Having another child may represent a very high cost for women who already have several children, but for women with fewer children, the perceived disadvantages of modern methods may be greater. Similarly, in Armenia and Jordan, the increase in withdrawal use with increasing age may result from a decrease in the perceived risk of fecundity, such that the ease of using withdrawal outweighs the calculated costs of other methods.

These individual factors for each couple are likely further affected by the specific healthcare landscape of a country including availability of contraceptive goods and services, dissemination of accurate and complete information about contraception, and affordability of contraceptives. These differ by country and are reflected in some of the associations between withdrawal use and education, wealth, and urban/rural residence detailed above. Finally, the existence of abortion services, as an additional available birth control option, also likely impacts a couples’ decision to use withdrawal. Abortion is integrated in the family planning strategy of Albania and widely available in Armenia as a former Soviet Republic that followed the Russian legalization model ^28^. In Turkey, previous studies have found that withdrawal failure is one of the primary reasons behind abortions ^17^. These contextual factors not only differ by country, but also likely differ in their weight on couples decisions, explaining the variety noted in the factors found to be associated with withdrawal across these four countries. Thus, while our study is limited to covariates available in DHS data, the findings point to a calculus of method choice by couples that will require temporal data of greater complexity to be further investigated.

While the study findings are limited by the cross-sectional design of these surveys, these are typically considered sufficient for an initial risk factor analysis. Unfortunately, data availability for countries in these regions is small preventing an exhaustive examination of the issue. Furthermore, as detailed previously, this analysis relies on surveys of female respondents about a male method which may affect the contraceptive distribution; while most of the correlates included are characteristics of women or household, the partners’ age and education level were reported by women and their accuracy may be limited. Finally, public data from these surveys tend to record only the most effective method reported. However, withdrawal can often be used in combination with other methods, such as rhythm or condoms.

These four countries have reached low levels of fertility with withdrawal as a, if not the, main method of contraception. Despite this, there remains an unmet need for contraception with the reported levels of mistimed or unintended births. Given that the current method mixes figure withdrawal use prominently, enabling every woman to achieve her fertility intentions relies, in part, on addressing barriers to modern contraception. To that end, programs can focus their attention on the correlates of withdrawal use. In general, reducing inequalities in women’s education to increase availability of information and improving decentralized access to affordable methods should provide women with modern methods and diversify the method mix. However, the absence of a single explanation for the prevalence of withdrawal in all these countries underlines the importance of considering the specific contexts in designing those efforts.

## Data availability

Demographic and Health Surveys Program.

URL: https://dhsprogram.com/data/available-datasets.cfm

This project contains the following underlying data:

- ALIR71FL.DTA (Individual Recode Stata dataset for Albania Standard DHS 2017–18)
- AMIR72FL.DTA (Individual Recode Stata dataset for Armenia Standard DHS 2015–16)
- JOIR73FL.DTA (Individual Recode Stata dataset for Jordan Standard DHS 2017–18)
- TRIR62FL.DTA (Individual Recode Stata dataset for Turkey Standard DHS 2013)

Data are available under the terms of the Creative Commons Zero "No rights reserved" data waiver (CC0 1.0 Public domain dedication).
