# Supplementary material for: Explaining withdrawal’s persistence: correlates of withdrawal use in Albania, Armenia, Jordan, and Turkey observed in a cross-sectional study
Source: Gates Open Res. 2021 Jun 9;5:92. [Version 1] doi: 10.12688/gatesopenres.13295.1 (PMC8313849; doi:10.12688/gatesopenres.13295.1)
Supplement: Peer review history

# Peer review: Reviewer response for version 1

**DOI:** 10.21956/gatesopenres.14535.r30762

**Published:** 2021-07-26

**Authors:** Amir Erfani

**Affiliations:** 1 Department of Sociology and Anthropology, Nipissing University, North Bay, ON, Canada

As a historical, natural contraceptive method, withdrawal has played a significant role in the fertility transition of Developed and Developing countries. A prime recent example is the case of Iran, where in the absence of an official family planning program right after the 1979 Islamic Revolution, urban and educated women controlled their births mostly by using coitus interruptus. As the present research signified, this method traditionally has been widely used in countries located in the Eastern Europe and Western Asia. Studies based on survey data, like the current research, usually provide contradictory explanations about the determinants of withdrawal use, suggesting different dynamics and contexts of withdrawal use. What is really missing in all these studies is a theoretical framework that can provide a general, universal explanation for the rationales behind choosing withdrawal over modern methods, while adopting a life course approach that explains determinants of withdrawal use over reproductive life course.

 This research could have benefited from adopting a theoretical framework that could direct the authors in selecting relevant theoretical covariates of withdrawal use, despite the limitation of variables in DHSs. The existing literature, mostly from qualitative studies, could provide a nice conceptual framework for determinants of withdrawal use.

 The authors noted that “Demographic and socioeconomic covariates typically associated with family planning use were included” (P.6). The selected common socioeconomic covariates hardly can measure directly contraceptive goals, evaluation, competence and access, as the major determinants of contraceptive use. There are some other relevant covariates in DHSs that can measure fertility intentions, contraceptive knowledge, contraceptive history and experience and access that are missing in this research.

 Moreover, the logic behind adopting two dependent variables has not been explained in this manuscript. As illustrated in the multivariate models, the results are different when the reference groups of the dependent variables become different: “none contraceptive users” vs. “non withdrawal users”. It is very important that the authors add a justification for why they have chosen these two different samples, and the two dependent variables, and their implications.

 The paper contributes to the underdeveloped literature, examining the determinants of withdrawal use.

Is the work clearly and accurately presented and does it cite the current literature?

Yes

If applicable, is the statistical analysis and its interpretation appropriate?

Yes

Are all the source data underlying the results available to ensure full reproducibility?

Yes

Is the study design appropriate and is the work technically sound?

Yes

Are the conclusions drawn adequately supported by the results?

Yes

Are sufficient details of methods and analysis provided to allow replication by others?

Yes

Reviewer Expertise:

Fertility, family planning and reproductive health, family transformation

I confirm that I have read this submission and believe that I have an appropriate level of expertise to confirm that it is of an acceptable scientific standard, however I have significant reservations, as outlined above.

# Peer review: Reviewer response for version 1

**DOI:** 10.21956/gatesopenres.14535.r30761

**Published:** 2021-06-25

**Authors:** Akbar Aghajanian

**Affiliations:** 1 Department of Sociology, Fayetteville State University, Fayetteville, NC, USA

The regional approach toward understanding the popularity of withdrawal even where modern contraceptives are easily available is innovative.

 Withdrawal is very popular in West Asian countries. For the Middleastern Countries, it is even much more popular in the urban areas where highly educated couples prefer it to the pill.

 This article adds to the limited literature in these areas.

Is the work clearly and accurately presented and does it cite the current literature?

Yes

If applicable, is the statistical analysis and its interpretation appropriate?

Yes

Are all the source data underlying the results available to ensure full reproducibility?

Yes

Is the study design appropriate and is the work technically sound?

Yes

Are the conclusions drawn adequately supported by the results?

Yes

Are sufficient details of methods and analysis provided to allow replication by others?

Yes

Reviewer Expertise:

Demography

I confirm that I have read this submission and believe that I have an appropriate level of expertise to confirm that it is of an acceptable scientific standard.

# Peer review: Reviewer response for version 1

**DOI:** 10.21956/gatesopenres.14535.r30760

**Published:** 2021-06-16

**Authors:** John A. Ross

**Affiliations:** 1 Independent Demographic Researcher, New Paltz, NY, USA

This interesting study of withdrawal use in four countries shows its range in popularity and in differentials by personal characteristics, in what amounts to four case studies. Contrasts go from the extreme case of Albania, perhaps the only country where a single method accounts for nearly all contraceptive use, to the case of Turkey with its high CPR and diminishing share of withdrawal use as modern use has risen. The risk ratios for use of withdrawal and other methods across the set of socio-economic variables are the core of the article, and these are discussed in detail in the text.

 It would help to provide briefly the historical setting for withdrawal use in these countries. Based on the UN compilation of all surveys, in Albania withdrawal's share after 2000 rose from 55% to 92%, while the CPR fell suddenly from 69% to only 42% between the 2008 and 2017 surveys. In Armenia the share was U-shaped, rising to 53% then falling to 43%. In Jordan the withdrawal share rose from about 10% in the 1970s and 1980s, growing steadily to 25% in 2017. In Turkey the use of withdrawal stabilized at about 25% of married women by the early 1980s (though only 20% in the 2018 DHS survey), while losing share (mix) from about half of all use to below a third by 2013 (29% in 2018).

 So while withdrawal was popular in these countries it's role was changing.

 Figure 1 doesn’t quite work visually due to the erratic trends, especially in black and white. However if the obvious outliers are neglected it shows that the withdrawal share is either increasing or is stable in most countries. A better way to communicate that is needed. (Remove Cambodia).

 Notable outcomes from the risk ratios (Table 4) are that larger family sizes elevate the use of both withdrawal and that of “any other method” in all four countries, and even more so for the other methods than for withdrawal. Higher education also matters, again more so for the use of other methods than for withdrawal. (Going by the partner’s education sharpens that contrast, with some irregularity by country). Household wealth shows uneven patterns, except for the remarkably high ratios in Turkey with more wealth, high for withdrawal and even higher for all other methods.

 Table 5 concerns the mix, i.e. a preference for withdrawal over that of other methods. The results echo some of those in Table 4 that show some dominance by other methods over withdrawal (in the contrasts between the top and bottom panels). In Table 5, withdrawal loses prominence with more living children and education and again in the wealthier households. 

Minor:

- It is regrettable that the 2018 Turkey survey tape was not available, but it is likely that the socio-demographic patterns would not vary enough to change the main conclusions about the differentials. 
- Some edits are needed, e.g. “women” rather than “users” on page 8 last paragraph, and elsewhere. In further editing it would help to intersperse more summary comments relieve the extensive text details.

Overall the article deserves indexing with revisions.

Is the work clearly and accurately presented and does it cite the current literature?

Partly

If applicable, is the statistical analysis and its interpretation appropriate?

Yes

Are all the source data underlying the results available to ensure full reproducibility?

Yes

Is the study design appropriate and is the work technically sound?

Yes

Are the conclusions drawn adequately supported by the results?

Yes

Are sufficient details of methods and analysis provided to allow replication by others?

Yes

Reviewer Expertise:

Demographic methods; family planning research; fertility analyses; developing countries.

I confirm that I have read this submission and believe that I have an appropriate level of expertise to confirm that it is of an acceptable scientific standard, however I have significant reservations, as outlined above.
